# Supplementary material for: Effect of the Addition of Humic Substances as Growth Promoter in Broiler Chickens Under Two Feeding Regimens
Source: Animals (Basel). 2019 Dec 9;9(12):1101. doi: 10.3390/ani9121101 (PMC6940755; doi:10.3390/ani9121101)
Supplement: Supplementary file 1 [file animals-09-01101-s001.pdf]

Supplementary Table 1. The relative intensity of the main band and associated functional groups of infrared spectra of the extract of humic substances (EHS) [1].

Wave number, cm^{-1}	Functional groups
3500-3250	OH extension of alcoholic and phenolic-OH, small contributions of NH_2, -CO-NH, S-H, and P-H
3000-2800	CH_3 and $-CH_2$ aliphatic groups (symmetric and asymmetric extension of -OH groups
1800-1600	C=O extension in lactones and NH_2 in amides - contribution of nitrates ($R-O-NO_2$), nitrites [R-O-NO)
1569	Pyridine derivatives - an extension of double rings
1408	CH_2 and CH_3 in aliphatic compounds, the vibration of bending CH_2, asymmetric deformation of CH_3
1210	C-O and OH of COOH, C-O of aryl-ethers and phenols
1118	C-O-C in aliphatic ethers, asymmetric extension
1025	Si-O-Si - asymmetric extension, C-O in polysaccharides and S=O in alkyl sulfoxides
848	Trisubstituted benzene, deformation of CH out of the plane, balancing of $Si-CH_3$
774	Multisubstituted benzene, C-Cl extension

[1] All spectra were collected in a range of 4000-400 cm^{-1}, with a resolution of 4 cm^{-1}, using 32 scans per sample
